# Flexural strength and surface hardness in polymethyl methacrylate provisional restorative material with zirconium nanoparticles: An *in vitro* study

**DOI:** 10.6026/973206300222737

**Published:** 2026-05-31

**Authors:** Rachamalla Srilekha, Ravi Kumar C, Chalapathi Rao D, Ravi Shankar Y, Sri Charitha L

**Affiliations:** 1 Department of prosthodontics, Mamata dental college, Khammam, Telangana, India

**Keywords:** Provisional polymethyl methacrylate resin (PPMMAR), zirconium nanoparticles, provisional restoration, flexural strength, surface hardness

## Abstract

Provisional PMMA restorations were not durable as they were deficient in flexural stability and surface hardness and their clinical relativity could not be trusted. Therefore, it is of interest to find out the ability of addition of 3 wt% zirconium nanoparticles to enhance mechanical properties of heat cured PPMMAR. It was tested on flexural strength on three-point bending test and surface hardness on Vickers hardness tester (160 of these). It was observed that zirconium reinforced specimens had a significantly high value of flexural strength and hardness than the control group (p < 0.05). Zirconium nanoparticles addition contributes to the mechanical behavior of PMMA to make it favourable in more permanent provisional restorations.

## Background:

A full course of prosthodontic and restorative treatment often relies on interim or provisional restorations. These restorations maintain function, occlusion and esthetics during the period between tooth preparation and placement of the definitive prosthesis. Temporary crowns and bridges must be carefully designed because they protect pulpal tissues, prevent tooth migration, maintain periodontal health and support phonetics and mastication while also providing the patient with an approximation of the final restoration [1]. Temporary restorations may remain in the oral cavity for extended periods, particularly during implant-supported or staged rehabilitative procedures. During this time, provisional materials must withstand the oral environment and functional occlusal forces while maintaining their form and purpose. Therefore, ideal provisional materials should allow ease of fabrication, adequate mechanical strength and dimensional stability to ensure clinical longevity [2]. Commonly used materials include polyethyl methacrylate (PEMA), bis-acryl composite resins and polymethyl methacrylate (PMMA).PEMA produces less exothermic heat during polymerization but demonstrates relatively low mechanical strength. Bis-acryl resins are rigid and can be conveniently fabricated chairside. However, PMMA continues to be widely used for long-span or long-term provisional restorations because of its flexibility, esthetics, affordability and reliable clinical performance. Heat-cured polymethyl methacrylate resin (PMMA) has been valued for its esthetic properties, color stability, wear resistance, repairability and ease of fabrication. Nevertheless, it has certain clinical limitations, including polymerization shrinkage, brittle behavior and susceptibility to functional stresses that may lead to fracture or marginal cracks. In particular, reduced flexural strength may result in fracture under occlusal loading, while inadequate surface hardness can increase material wear [3]. To overcome these limitations, reinforcement methods such as the incorporation of glass or polyethylene fibers and various chemical additives have been proposed. However, these approaches may compromise esthetics and complicate material handling [4]. In recent years, nanoparticle reinforcement has gained considerable attention. Due to their extremely small size and large surface area , nanoparticles can improve stress distribution within the resin matrix and help inhibit crack propagation[5]. Among these, zirconium oxide (ZrO_2_) nanoparticles show significant promise because of their high fracture toughness, hardness and excellent biocompatibility. Their color and chemical stability make them compatible with PMMA and their incorporation has been reported to improve flexural strength and surface wear resistance through mechanisms such as crack deflection and stress transfer within the resin matrix [6]. Although several studies have evaluated zirconia-reinforced PMMA, most investigations have focused on self-cure or CAD/CAM provisional materials. Limited data are available regarding heat-cured PMMA systems [7]. Therefore, it is of interest to evaluate the effect of incorporating zirconia nanoparticles on the flexural strength and surface hardness of heat-cured PMMA, thereby assessing its potential suitability as a durable long-term provisional restorative material.

## Materials and Methods:

This was an *in vitro* study carried out in the Department of Prosthodontics, Mamata Dental College, Khammam, Telangana, India. It was approved by the Institutional Ethics Committee.

## Grouping of samples:

A total of 160 samples were fabricated and divided into two main groups. Each group consisted of 80 samples, further subdivided into two subgroups, each containing 40 samples (Table 1).

## Study procedure:

The materials used were heat polymerised provisional acrylic resin, zirconium oxide nanoparticles and dental stone. Three brass metal dies of dimensions 65 mm in length, 10 mm in width and 3 mm in height were made (ISO 1567 standard) for the fabrication of test samples.

## Fabrication of specimens:

For the control group (Group I), 40 specimens of conventional heat-cured PPMMAR (65 x 10 x 3 mm) were fabricated using metal molds invested in dental stone within brass flasks. A separating medium was applied before pouring the stone mixed in a vacuum mixer. After setting, 5 g of PPMMAR powder was mixed with 3.5 ml monomer to the dough stage, packed into the molds and the trial was closed. Final pressing was done at 1500 psi, followed by polymerization at 74°C for 2 hours and 100°C for 1 hour. This short curing cycle minimized porosity and ensured a uniform polymer matrix. Specimens were deflasked, finished with tungsten carbide burs and sandpaper and stored in distilled water. For the test group (Group II), 80 specimens were fabricated by incorporating 3 wt% zirconium oxide (ZrO_2_) nanoparticles (3 g per 100 g of polymer) into PPMMAR. Nanoparticles were first mixed with the polymer using a mortar and pestle and further blended by tumbling. The modified powder was then processed identically to the control group. Specimens were finished and polished as before, ensuring standard dimensions and preserved in distilled water.

## Testing for flexural strength:

Flexural strength testing was performed using an Instron Universal Testing Machine. A total of 80 samples, 40 from each group (unmodified and modified PPMMAR), were tested. All specimens were tested in a load cell with a 250-kilo Newton capacity. A vertical force was applied at the specimen's midpoint by a chisel, at a cross-head speed of 5 mm/min and a span length of 40 mm, until specimen fracture occurred under flexural loading. Fracture was indicated by a sharp crackling sound accompanied by a sudden drop in the digital signal. The load required to induce failure was recorded in MPa (N/mm^2^). Results were recorded using a three-point bending test, with readings noted and tabulated.

## Testing for hardness:

Hardness testing was performed using the Vickers method, an optical measurement system outlined in ASTM E-384. This microhardness test employs a diamond indenter with light loads to create small indentations, which are optically measured and converted into hardness values. A square-based pyramidal diamond indenter was used for testing. The method is versatile for various materials but requires highly polished specimen surfaces to ensure accurate and consistent indentation measurements. To achieve reliable results, samples must be mounted in a plastic medium, with the surface held perpendicular to the indenter. Proper surface preparation ensures uniform indentation geometry and precise measurement. Larger indentations are preferred to enhance measurement resolution.

## Statistical analysis:

Statistical analyses were performed using the Statistical Package for Social Sciences (SPSS) for Windows, version 26.0. Descriptive analysis included the expression of flexural strength and surface hardness in terms of mean and standard deviation (SD). The mean and SD scores for flexural strength and surface hardness were obtained and compared across different study groups using the One-way ANOVA test. Multiple comparisons within different study groups were conducted using an independent t-test. The statistical significance p-value was set at P < 0.05.

## Results:

The maximum flexural strength mean value (70.50 MPa) was seen in Group II- PPMMAR + 3wt% zirconium oxide (test group) and the minimum flexural strength mean value (53.48 MPa) was seen in Group I- Control group - unmodified PPMMAR (Table 2). An independent t-test compared the flexural strength of conventional PPMMAR (control) and ZrO_2_ nanoparticle-incorporated PPMMAR (test) groups, each with 40 specimens. The test group showed a significantly higher mean flexural strength (70.50 MPa) than the control group (53.48 MPa), with a t-value of 9.2015 and a p-value of 0.0001.

The statistically significant difference confirms that the incorporation of zirconium oxide nanoparticles effectively enhances the flexural strength of PPMMAR (Table 3). Maximum surface hardness mean value (29.12 VH) was seen in Group II- PPMMAR + 3wt% zirconium oxide (test group) and minimum surface hardness mean value (23.22 VH) was seen in Group I- Control group - unmodified PPMMAR (Table 4). An independent t-test comparing surface hardness between control and ZrO_2_ nanoparticle- incorporated PPMMAR groups (n=40 each) showed a significant increase in the test group. The control group had a mean surface hardness of 23.22, while the Zr test group recorded 29.12. The t-value was -20.1188 with a statistically significant p-value of 0.0001 (Table 5).

## Discussion:

Provisional restorations play a crucial role in prosthodontic and restorative dental practice, as they help maintain function, occlusal stability and esthetics during the transitional phase before definitive prosthesis placement. These restorations protect pulpal tissues, prevent tooth migration, support periodontal health and assist in phonetics, mastication and esthetic evaluation during treatment [1].Provisional restorative materials should possess favorable mechanical properties, particularly high flexural strength and surface hardness, to ensure clinical durability. These properties are essential for withstanding occlusal forces, providing adequate wear resistance and preventing marginal breakdown or microleakage during the interim period [8]. Common materials used for provisional restorations include polyethyl methacrylate (PEMA), bis-acryl composite resins and polymethyl methacrylate (PMMA) [9]. Among these, PMMA has been widely used since the 1930s due to its affordability, biocompatibility and acceptable esthetic properties. However, its clinical performance in the oral environment is limited by certain shortcomings such as relatively low flexural strength and inadequate surface hardness. Provisional prostheses are subjected to complex masticatory forces and if the material lacks sufficient strength and toughness, it may result in fracture or reduced service life. Flexural strength, defined as the maximum stress a material can withstand during bending, is a critical factor in preventing prosthesis failure. Repeated low-intensity masticatory forces can initiate and propagate microcracks in PMMA, gradually reducing its functional lifespan [10]. Surface hardness is another important characteristic because it reflects the material's resistance to abrasion and wear. Reduced hardness may lead to surface roughness, increased plaque accumulation, staining and eventual deterioration of esthetics. The Vickers microhardness test is commonly used to evaluate this property in dental materials [11]. To overcome these limitations, several modifications have been explored, including copolymerization and reinforcement with metallic oxide fillers. Among these, zirconium oxide (ZrO_2_) nanoparticles have emerged as a promising additive due to their excellent biocompatibility, high fracture toughness and ability to enhance the mechanical properties of PMMA. Nanotechnology, which involves the manipulation of materials at the nanometer scale, has significantly advanced restorative dentistry. The incorporation of nanofillers can enhance both the mechanical and optical properties of dental materials. Various nanoparticles such as silica (SiO_2_), zinc oxide (ZnO), titanium dioxide (TiO_2_) and zirconium oxide (ZrO_2_) have been investigated for strengthening polymer-based restorative materials. TiO_2_ nanoparticles may increase opacity but can also cause undesirable discoloration due to intense light scattering and high refractive index. SiO_2_ generally has minimal effect on color, whereas ZnO provides moderate brightness with minor color alteration. Zirconia nanoparticles tend to enhance whiteness and opacity but may reduce translucency, which can influence esthetic outcomes. Therefore, appropriate concentration and uniform dispersion are critical to maintaining a balance betweenmechanical reinforcement and esthetic properties. Due to their nanoscale size and large surface area, these particles enhance cross-linking within the resin matrix and improve the overall structural integrity of the material [12].

Zirconia nanoparticles are particularly advantageous in dental applications because they are white, esthetically compatible, mechanically strong and biocompatible. Their incorporation into PMMA can inhibit crack propagation within the resin matrix, thereby increasing fracture toughness and enhancing the long-term durability of provisional restorations [13]. [14]. In the present study, PMMA samples were prepared under two conditions: A control group consisting of conventional PMMA and a test group containing PMMA reinforced with 3 wt% zirconium oxide nanoparticles. All specimens were fabricated using a constant polymer-to-monomer ratio and standardized polymerization cycle (74°C for 2 hours followed by 99°C for 1 hour) in accordance with ISO 1567:1999 standards. Flexural strength was evaluated using a Universal Testing Machine (UTM), while surface hardness was assessed using the Vickers microhardness test. Flexural strength testing was performed on 80 specimens. The control group (Group IA) demonstrated a mean flexural strength of 53.48 ± 3.59 MPa, whereas the zirconia-reinforced group (Group IIA) showed a significantly higher mean value of 70.50 ± 8.20 MPa. Statistical analysis indicated a significant improvement in flexural strength following the addition of zirconia nanoparticles (p = 0.001). Similarly, surface hardness evaluation was conducted on 80 specimens. The control group (Group IB) exhibited a mean surface hardness value of 23.22 ± 1.30, while the zirconia-reinforced group (Group IIB) demonstrated a significantly higher value of 29.12 ± 1.33. The difference between the groups was statistically significant (p = 0.001). These results align with the findings of study about the effects of treated zirconium oxide nanoparticles at concentrations of 1 wt%, 2.5 wt% and 5 wt% on provisional prosthetic materials. The study reported a concentration-dependent increase in surface hardness, with significant improvements observed at 2.5 wt% and 5 wt%. This enhancement was attributed to improved dispersion and integration of nanoparticles within the polymer matrix, leading to increased cross-link density and enhanced resistance to surface indentation and wear [15]. Despite the promising findings, certain limitations must be acknowledged. This study was conducted under *in vitro* conditions, which do not fully replicate the complex and dynamic oral environment. The use of flat test specimens may not accurately simulate the anatomical contours and stress distribution encountered in clinical prosthodontic restorations. Additionally, the long-term performance of zirconium oxide nanoparticles within the resin matrix was not evaluated. The loading protocol used in this study also did not fully reproduce the magnitude, direction and variability of masticatory forces present intraorally, which may limit the clinical extrapolation of the results. Furthermore, scanning electron microscopy (SEM) analysis was not performed to assess nanoparticle dispersion, adhesion, or interfacial bonding within the PMMA matrix.

## Conclusion:

We show that the addition of 3wt% zirconium oxide nanoparticles was able to substantially increase the mechanical properties of provisional polymethyl methacrylate resin. To be precise, the hardened PPMMAR showed a significant increase in the flexural strength and surface hardness relative to the unaltered control group, which pointed to the fact that the reinforcement of the material with zirconium oxide can enhance its service life and performance in the clinical setting.

## Figures and Tables

**Table 1:** Sample grouping

	**Subgroup**	**Material Composition**		**Number of Samples**
Group I (Control)	Unmodified PPMMAR (F.S.)	Heat-cured PPMMAR without ZrO_2_	Flexural Strength	40
	Unmodified PPMMAR (S.H.)	Heat-cured PPMMAR without ZrO_2_	Surface Hardness	40
Group II (Study)	Modified PPMMAR (F.S.)	PPMMAR with ZrO_2_ nanoparticles	Flexural Strength	40
	Modified PPMMAR (S.H.)	PPMMAR with ZrO_2_ nanoparticles	Surface Hardness	40
Total				160

**Table 2:** Comparison of mean flexural strength values between different groups (control group and Zr test group) by ANOVA test

	**N**	**Mean**	**SD**	**SE**	**95% CI for mean**		**Min**	**Max**
					**Lower Bound**	**Upper Bound**		
Control (Group-I)	40	53.48	3.59	0.57	51.14	53.43	46.49	58.11
Test (Group-II)	40	70.5	8.2	1.3	67.88	73.12	54.65	80.04

**Table 3:** Comparison of two groups (Control group and Zr test group) with mean Flexural strength (MPa) by an Independent t-test

**Group**	**n**	**Mean**	**SD**	**SE**	**t-value**	**P-value**
Control (Group-I)	40	53.48	8.35	1.32	9.2015	0.0001*
Test (Group-II)	40	70.5	8.2	1.3		

**Table 4:** Comparison of mean surface hardness values between different groups (control group and Zr test group) by ANOVA test

**Group**	**N**	**Mean**		**SE**	**95% CI for mean**		**Min**	**Max**
					**Lower Bound**	**Upper Bound**		
Control (Group-I)	40	23.22	1.3	0.21	22.8	23.64	21.03	25.76
Test (Group-II)	40	29.12	1.33	0.21	28.7	29.55	26.86	30.86

**Table 5:** Comparison of two groups (Control group and Zr test group) with mean Surface hardness values by an Independent t-test

**Group**	**n**	**Mean**	**SD**	**SE**	**t-value**	**P-value**
Control (Group-I)	40	23.22	1.3	0.21	-20.1188	0.0001*
Test (Group-II)	40	29.12	1.33	0.21		

## References

[1] Alrais S (2026). Sci Rep..

[2] Gad MM (2021). J Prosthodont..

[3] Abushowmi TH (2020). J Prosthodont..

[4] Zidan S (2020). Int J Nanomedicine..

[5] Albasarah S (2021). J Adv Prosthodont..

[6] Qaw MS (2020). J Prosthodont..

[7] Li GH (2021). Mech Behav Biomed Mater..

[8] Hamdy TM (2024). BMC Oral Health..

[9] AlQahtani M, Haralur SB (2020). Medicina (Kaunas)..

[10] Vivek R (2025). J Pharm Bioallied Sci..

[11] Elzahar HB (2022). Int J Nanomedicine..

[12] Deb S (2020). J Contemp Dent Pract..

[13] Alhotan A (2021). Materials (Basel)..

[14] Özatik S, Alan CB (2024). J Oral Sci..

[15] Kaur H (2022). J Contemp Dent Pract..

